# Search for blood or water is influenced by *Borrelia burgdorferi* in *Ixodes ricinus*

**DOI:** 10.1186/s13071-014-0526-2

**Published:** 2015-01-06

**Authors:** Coralie Herrmann, Lise Gern

**Affiliations:** Institute of Biology, Eco-Epidemiology Laboratory, University of Neuchâtel, Emile-Argand 11, 2000 Neuchâtel, Switzerland

**Keywords:** *Ixodes* ticks, Phenotypic traits modification, Vector manipulation, Water balance, Blood meal, *Borrelia burgdorferi*

## Abstract

An increasing number of studies suggest that vector-borne parasites are able to alter phenotypic traits in their arthropod vectors so that microorganism transmission is enhanced. This review documents this phenomenon, which occurs between *Borrelia burgdorferi* bacteria, the causative agents of Lyme borreliosis, and their tick vectors belonging to the *Ixodes ricinus* complex. It also reviews the influence of other tick-borne pathogens on these ticks. Ticks belonging to the *Ixodes ricinus* complex benefit from *Borrelia* infection by an increased lifespan (more fat and more resistance to desiccation) and by an increased questing period (less need to move to the litter zone to rehydrate), which enhances tick chances to find a host and to subsequently transmit the pathogens.

## Background

**Figure 1:**
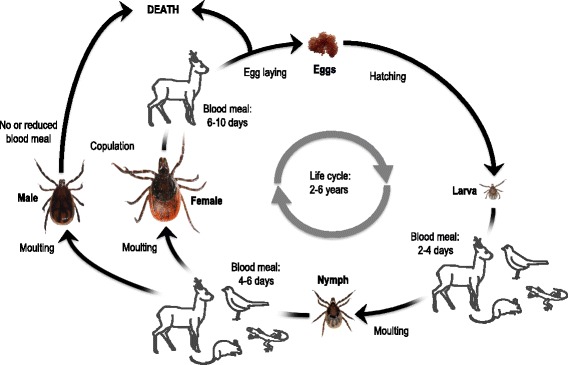
***I. ricinus*** **life cycle.** *I. ricinus* is a three-host tick of which each stage, i.e. larva, nymph and adult female (adult males may take a small blood meal but do not fully engorge), feeds on a different vertebrate host. *I. ricinus* ticks parasitize a wide range of hosts, i.e. more than 300 different vertebrate species [9]. Immature ticks particularly infest rodents such as *Apodemus flavicollis*, *Myodes glareolus* and *Sciurus vulgaris* [32,70-73]. Immature ticks also feed on ground-foraging birds (such as *Turdus* spp), lizards and artiodactyls [9,74]. Adults mainly feed on larger mammals, but are usually outnumbered by immature ticks on such hosts [75]. *I. ricinus* does not show host specificity and the most important determinants of host choice are host habitat and behaviour, microclimate conditions [32], and questing height of the different tick stages. Larvae and nymphs usually stay closer to the ground, i.e. they are predominantly observed between 0 to 30 cm and 30 to 70 cm, respectively [76,77], probably because they are more sensitive to ambient humidity than mature stages due to their high surface area to volume ratio [32]. Male and female adults are observed higher up on the vegetation, usually 60 to 80 cm above the ground, but they may be found higher than 1.5 m depending on the surrounding vegetation [76,77] (Tick pictures by N. Tonetti; Host pictures by P.-F. Humair and L. Gern).

The idea that a parasite can modify the phenotype of its host by changing the host perception of the environment and/or behaviour in order to complete its transmission cycle is intriguing. This phenomenon is well established and documented in hundreds of distinct host-parasite associations in all major phyla of living organisms [1,2]. While most of the known cases involve only subtle modifications in host phenotypes, some are doubtlessly spectacular. For vector-borne pathogens, the best-known examples involve pathogens transmitted by blood-sucking insects taking short blood meals on multiple hosts such as mosquitoes, sand flies, or tsetse flies. In such cases, manipulation usually consists of modifying vector behaviour so that the number of bitten hosts (and thereby infected hosts) is increased using strategies such as higher biting rates, shortened blood meals, a longer lifespan, etc. [3-5]. Unlike haematophagous insects, hard ticks feed for days (Figure 1) [6] and transmission of tick-borne parasites often needs some time after the beginning of tick attachment to occur (i.e. several hours), as for example the transmission of the agents of Lyme borreliosis (*Borrelia burgdorferi sensu lato* (s.l.)) by the European vector *Ixodes ricinus* [7,8]. Unlike insect-borne parasites, tick-borne parasites cannot increase the number of hosts encountered by their vectors during their life cycle (Figure 1) and other mechanisms have to intervene. Moreover, although finding a host is crucial for any haematophagous arthropod, ticks such as *I. ricinus* have an additional need that governs their behaviour and life cycle: maintaining their water balance. This need is particularly important for ticks because their parasitic life is very short (three blood meals, each of them taken over a few days) compared to their lifespan that extends over many years, spent in the environment (Figure 1). Maintaining a water balance is not compatible with host-finding behaviour and is even detrimental to host-finding success. Therefore tick-borne parasites could be expected to influence tick behaviour in such a way as to maximise host-finding success in order to be transmitted.

## Review

### The search for blood

*I. ricinus* is a generalist tick that feeds on more than 300 different vertebrate species ranging from mammals to reptiles or birds [9]. It finds its vertebrate host while questing, i.e. waiting on low vegetation with its first pair of legs outstretched in the air. When a vertebrate host brushes the spot where the tick is waiting, the arthropod grabs the passing host, climbs onto it, and takes a blood meal. While questing for a host, ticks are often exposed to unfavourable moisture conditions and they frequently interrupt their questing, and move to the litter zone. There they are quiescent in a humid atmosphere where they rehydrate [10]. This behaviour shortens questing periods and thereby reduces tick’s chances to find a host and transmit microorganisms.

### The search for water

*I. ricinus*, like other terrestrial arthropods, needs to maintain its water balance in an environment whose relative humidity (RH) is often below its comfort threshold (situated between 86 to 96% RH according to Lees [11]). When RH is below this threshold, the tick needs to minimise water loss and maximise active uptake of water vapour from the atmosphere [12]. *I. ricinus* loses water through the integument of its body surface when it is exposed to relatively dry conditions (transpiratory loss) [12] such as those that *I. ricinus* experiences on vegetation while questing for a host. In addition, *I. ricinus* loses water in the course of respiratory exchange via the tracheal system (respiratory loss) [12]. This occurs during mobility – when the tick ascends the vegetation to find a questing spot and later descends from the vegetation to reach a moister environment – since respiration increases when ticks move [13-15]. Water loss due to transpiration and respiration is compensated for by acquiring water from the environment. *I. ricinus* does not drink liquid water [11,16,17], but rather extracts water vapour from the atmosphere [11,12,18], in particular by active sorption [19]. In nature, the tick achieves this by periodically returning down to moist surroundings such as the litter layer [10,11], as shown by dehydrated *I. ricinus* nymphs moving preferentially towards fully saturated air under laboratory conditions [18,20].

Perret *et al.* [21] reported that the duration of questing depends on desiccating conditions: when conditions are less desiccating, ticks quest for longer periods before moving to the litter zone, which increases their chances to find a host. In experimental settings locomotion occurs primarily during darkness in both *I. scapularis* [22] and *I. ricinus* [21]. Such behaviour has been considered to be a means to minimise water loss (and energy costs to reabsorb it) by undertaking locomotion during less desiccating conditions coinciding with darkness [21].

### Seasonal questing activity

**Figure 2:**
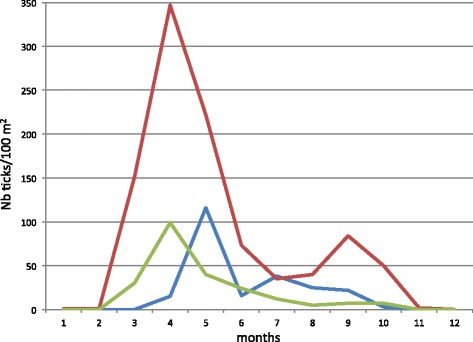
**Stylised** ***I. ricinus*** **seasonal questing activity (based on data collected in the Neuchâtel area, Switzerland).** In Switzerland, questing ticks may be collected as early as mid-February to early March [35]. In fact, questing *I. ricinus* ticks are active when the daily maximal temperature has reached 7°C over 5 days [29]. Adults and nymphs usually emerge first, followed by larvae. Questing tick density increases progressively as weather conditions get warmer [29] until peak density is recorded in spring, usually between April and June [34]. Questing density then decreases gradually due to decreasing numbers of unfed ticks still seeking a host and to increasingly drier weather conditions [29,31], so that *I. ricinus* ticks rarely quest during summer, except at higher altitudes where the climate is milder [33,34]. In autumn, when favourable conditions of temperature and humidity are back, a second peak of questing ticks may be observed [34]. However, the autumn peak is of lower intensity than the one observed in spring and is absent if weather conditions are unfavourable [29,31,34]. The last questing *I. ricinus* ticks are usually sampled in October or early November [34] as ticks return progressively to an inactive state during winter [26,78]. Larvae: green; nymphs: red; adults: blue.

Temperature, humidity and photoperiod shape *I. ricinus* behaviour and lifespan in nature. Hence, the host-finding activity of *I. ricinus* is greatly influenced by weather conditions and progress is governed by a seasonal pattern (Figure 2). During winter, ticks do not usually quest and are in the leaf litter or in the upper layers of the soil where temperatures are milder than on the vegetation [23,24]. The interaction between photoperiodicity and temperature appears to determine whether *I. ricinus* ticks are active or not during winter [25-27]. However, additional research is needed to disentangle the roles played by photoperiodicity and temperature on the winter physiology/behaviour of *I. ricinus. I. ricinus* usually displays questing activity from February-March to September-October, but importantly this depends on temperature [28]. In spring, *I. ricinus* usually quests for a host when temperature ranges between 7 and 24°C in Switzerland [28,29]. During warmer and drier months (i.e June, July and August), questing tick activity is reduced while it may reappear in autumn (Figure 2). This is mainly due to a new cohort of ticks that emerge [30] but is also partly due to ticks from the previous cohort that quit quiescence [21,29,31]. Variability in air humidity also regulates tick seasonal questing activity. It is known that *I. ricinus* displays little resistance to desiccation [11,28] and that RH needs to be above 70 to 80% to allow questing activity and survival [28]. Saturation deficit (SD) (a measure of the drying power of the atmosphere that depends on both temperature and RH [32]), influences the seasonal activity of *I. ricinus*. SD values that are lower than 5 mmHg are favourable for *I. ricinus* questing activity and development, [21,33]. In fact, *I. ricinus* questing activity [33-35], duration of questing [21,31], and survival in nature [29,31] are reduced when SD values are high. *I. ricinus* larvae are less tolerant of desiccation and therefore die earlier than nymphs under desiccating conditions [28]. In turn, *I. ricinus* nymphs are more affected by desiccating conditions (therefore suffering a higher death rate) than adults, as shown by the partial restoration of the questing adult population (but not of the questing nymph population) after each drought event in nature [31]. The lower resistance to desiccation observed in immature stages compared to adult stages [11,28,31] is probably due to their smaller size, lower water content and a higher surface area to volume ratio (causing a proportionally higher water loss) than in adults [32]. Tick populations are most importantly exposed to high SD values in spring (the usual questing activity peak in continental Europe) and summer, and to a lesser extent in autumn. Accordingly, *I. ricinus* questing density is low when SD is high during spring and summer [29].

### Influence of climate on the geographic distribution of *I. ricinus*

Climate change affects *I. ricinus* geographic distribution, as illustrated by the shifts observed during the last decade towards higher latitudes in its northern distributions in Sweden [36,37] and Norway [38], and towards higher altitudes in the Czech Republic [39-41], Switzerland [34] and Norway [38]. Higher temperatures in spring and autumn extend the vegetation period and the season during which *I. ricinus* develops, allowing vegetation communities (and therefore vertebrates on which *I. ricinus* feeds) and tick populations to colonise and establish at higher latitudes [42] and altitudes [34,43]. In addition, climate change affects *I. ricinus* populations in areas where the tick is already established, notably influencing its seasonal questing activity [27,29], and seems to gradually extend *I. ricinus* seasonal activity as it uses milder winter periods for re-emerging and questing [26,27].

### Energy in ticks

Ticks consume energy (i.e. fat) while locating and ascending vegetation stems from which they quest for a host, and descending to moist conditions at the base of the vegetation where they restore their fluid content [10,12,16,19]. This energy is acquired through the single blood meal that is taken by each of the three developmental stages. Although the blood meal primarily consists of proteins (up to 95%), the digestive products obtained during feeding are largely converted to, and stored as, fat (lipids) [44]. Lipids are stored in epithelial cells of the midgut [45,46], and in the fat body of ticks (a diffuse organ of highly dispersed strands of cells adhering to the branches of the tracheal system and, occasionally, to other internal organs) [47]. As *I. ricinus* has no other energy sources, fat content declines over time between each blood meal [30,36,48]. Randolph and Storey [32] have demonstrated that ticks kept under dry conditions consume their fat twice as fast as those kept under wet conditions. In addition, a linear relationship between temperature and lipid consumption has been observed in male and female *I. ricinus* [49]. When weather conditions get drier [21,32], *I. ricinus* needs to move down the vegetation to rehydrate, more often consuming even more energy in the process. Hence, in *I. ricinus*, the rate of lipid consumption increases under unfavourable conditions of humidity [32] and temperature [49]. Accordingly, fat content reflects both the history of the tick stage and the energy reserves remaining for future use [30,32].

In summary, *I. ricinus* has two basic and interconnected needs that govern its entire behaviour and life cycle: finding a host for blood feeding and maintaining its water balance. Finding the right balance between these two needs, which are rarely in agreement, is a real challenge for *I. ricinus*. To fulfil these needs, the tick consumes energy when it climbs up the vegetation to quest, and when it periodically moves down the vegetation to rehydrate in the litter layer.

### Modification of phenotypic traits of ticks by microorganisms

Various studies have reported that bacteria and viruses may modify the traits of *I. ricinus, I. persulcatus, I. scapularis* and *I. pacificus;* the main tick species playing a role in the transmission of *B. burgdorferi* s.l. (Table 1). Even though it has been known for decades that *I. ricinus* is sensitive to humidity and that dehydrated ticks move preferentially towards a moister environment [18,20], the influence of infection on tick tolerance to desiccation has been poorly investigated. Alekseev [50] has reported that *I. persulcatus,* the Asian vector of *B. burgdorferi* s.l., infected with the tick-borne encephalitis virus (TBEv) choose a higher questing height. Similarly, other authors have shown that *Borrelia*-infected *I. scapularis* [51]*,* the North American vector of *B. burgdorferi* s.l., and *I. persulcatus* [52]*,* also choose a higher questing height, which exposes ticks to more desiccating conditions. This suggests that TBEv- and *Borrelia*-infected ticks are less sensitive to a dry environment than uninfected ticks. In fact, it has been shown recently that *Borrelia*-infected *I. ricinus* nymphs have a lesser need to move towards a moist environment, which is favourable for their water balance, than uninfected nymphs [53]. When they are given the choice between dry and moist environments *Borrelia*-infected individuals stay where the RH levels are between 70 and 75% (corresponding to SD values of 6.2 to 5.2 mmHg at room temperature, ~23°C), whereas uninfected ticks tend to move to a moister environment when SD reaches 4.4 mmHg (corresponding to 80% RH at 24°C) in nature [28]. In this case, vector manipulation consists of increasing the tick’s chances of finding a vertebrate host (and thereby transmitting the pathogen) by increasing questing time, i.e. by allowing the tick to quest for a longer time under desiccating conditions before returning to a moister environment, such as the litter layer, to rehydrate.

**Table 1:** **Phenotypic traits modified by tick-borne pathogens in the main vectors of** ***B. burgdorferi***

**Modified trait**	**Tick species** ^**a**^	**Stage** ^**b**^	**Pathogens** ^**c**^	**Detailed effect**	**Reference**
Questing activity	*Ip*	A	*Bb*	Walk shorter distances	[50,79]
	*Ip*	N, A	*Bb*	Questing activity is increased but inhibited more importantly by temperature	[80]
	*Ip*	N, A	*Bb*	Questing triggered by higher temperature and lower relative humidity	[66]
	*Ip*	A	*Bb*	Reach higher questing height, walk slower	[52]
	*Ip*	A	TBEv	Walk faster, reach higher questing height, more tolerant of desiccation	[50]
	*Ir*	L, N, A	*Bb*	Walk shorter distances	[81]
	*Ir*	N	*Bb*	Walk less, stay in a relatively dry environment	[53]
	*Ir*	A	TBEv	Walk faster, more tolerant to tick-repellent	[82]
	*Is*	N	*Bb*	Walk longer distances, reach higher questing height, attracted by vertical surfaces	[51]
	*Is*	A	*Bb*	Walk shorter distances, reach lower questing height, avoid vertical surfaces	[51]
Survival & energy reserves	*Ip*, *Ir*	A	*Bb*	Prolonged survival	[67]
	*Ir*	N, A	*Bb*	Increased survival under desiccating conditions	[56]
	*Ir*	N	*Bb*	Higher energy reserves (fat content)	[61]
	*Is*	N	*Ap*	Increased survival under cold conditions	[55]
	*Is*	N	*Bm*	Increased blood meal size	[63]

^a^ *Ip*, *I. persulcatus*; *Ir*, *I. ricinus*; *Is*, *I. scapularis.*

^b^L, larvae; N, nymphs; A, adults.

^c^ *Ap*, *A. phagocytophilum*; *Bm*, *B. microti*; *Bb*, *B. burgdorferi* s.l.; TBEv, TBE virus.

During winter months, ticks may be exposed to very cold weather and survival represents a challenge for them [54]. Interestingly, an increase in the survival under cold conditions of *I. scapularis* infected by *Anaplasma phagocytophilum* has been reported recently [55]. *A. phagocytophilum* has been shown to induce the production in ticks of an antifreeze glycoprotein protecting ticks from extreme cold. Similarly, extreme weather conditions during spring and summer involving high temperature and low RH not only influence questing activity [33-35] and duration of questing [21,31], but also survival of ticks in nature [29,31]. Recently, increased survival of *I. ricinus* under highly desiccating (hot and dry) conditions when infected by *B. burgdorferi* s.l. has been reported [56]. The mechanisms leading to this have not been determined. Nevertheless, it has been hypothesised that on the one hand, *Borrelia* spirochetes that are in the midgut and/or those that are in other tick organs [57,58] might change the physiology and/or metabolism of organs involved in water sorption, storage, or loss. Similarly to *A. phagocytophilum*, *Borrelia* spirochetes have also been described to change gene expression in ticks. In fact, Ramamoorthy *et al.* [59] reported the increased expression of one gene in *B. burgdorferi*-infected *I. scapularis* salivary glands during blood feeding. Expression modifications of genes involved in the maintenance of water in ticks would result in enhanced water storage in infected ticks, and the dependence of *I. ricinus* on humidity might therefore be reduced by *Borrelia* infection. It may also be hypothesized that spirochetes, which are known to change their gene expression according to temperature [60], might be able to modify tick gene expression under varying temperature conditions as well, inactivating genes that govern non-vital functions in the tick, slowing down metabolism in such a way that energy resources would essentially be devoted to resistance to unfavourable conditions, while maintaining favourable living conditions for spirochetes. Since it is known that spirochetes are non-infectious in unfed ticks [8] the switch-off of spirochete infection factors in unfed ticks might also trigger tick metabolism to slow down, explaining infected ticks better survival under challenging weather conditions. The chance of pathogen transmission is increased by better survival of infected ticks, giving the latter more time and opportunities to find a vertebrate host (and thereby transmit the spirochetes) under unfavourable conditions. On the other hand, these findings might be explained by modifications of tick behaviour by spirochetes, so that infected ticks take more risks by staying immobile under desiccating conditions, although usually *I. ricinus* ticks are known to show increased movement under such conditions [18,20]. Such risky behaviour would be beneficial to infected ticks, as they would spare energy reserves while uninfected ticks would deplete their reserves trying to find a moister environment without success, resulting in a higher death rate of more active uninfected ticks.

### Fat content in ticks

According to some of the aforementioned observations, *Borrelia*-infected ticks are more tolerant of desiccating conditions (with a lower need to rehydrate, better survival and increased questing time under these conditions) than uninfected ticks. Since high desiccation forces *I. ricinus* ticks to consume more energy by moving down the vegetation to rehydrate more frequently [21,29,32], the observed higher tolerance to desiccation in infected ticks could be due to their higher energy reserves (more fat). This has recently been observed; *I. ricinus* ticks that are infected by *Borrelia* contain more fat than uninfected individuals and thus have higher energy reserves [61]. For an average body size of 66.4 μg, the mean fat content of an infected tick is 12.1% higher than that of an uninfected one. Bioenergetic calculations have shown that *Borrelia* spirochetes consume a negligible fraction of the tick energy reserves to grow [61]. In fact, under anaerobic conditions the energy required to grow a median population of 3,410 spirochetes using glucose corresponded to 0.10% of the total fat reserves of the nymph. However, the study did not revealed how higher energy reserves in ticks and *Borrelia* infection are associated, as mechanisms causing this phenomenon are presumably multiple and interconnected. Hypotheses may nevertheless be formulated. Various processes may operate on tick-host interactions (quantity and quality of blood-meal). *Borrelia* spirochetes in the vertebrate host might suppress host immune responses resulting in increased blood meal size in the tick. This has been shown to occur in *I. trianguliceps* [62] and *I. scapularis* ticks [63] feeding on rodents infected by *Babesia microti*, a tick-borne protozoan known to be immunosuppressive (unlike *B. burgdorferi*). The increased blood meal size might be subsequently converted into more fat content in the tick. Spirochetes in the blood might change the quality of the blood by increasing glucose concentration in infected blood [64]. Alternatively, *Borrelia* spirochetes might influence tick physiology (blood digestion and/or moulting process) by changing the expression of genes involved in fat storage in ticks (such as the *4E-BP* genes described by Kume *et al.* [65]) so that fat storage is enhanced during/after the blood meal. Spirochetes may also alter the behaviour of ticks in a way that reduces energy consumption by reducing ticks movements in infected ticks [51,53,66]. Although causes leading to such a phenomenon (i.e. higher fat content in infected ticks) remain unknown, its consequences are easy to imagine. In all these cases, the pathogen increases its transmission chances by increasing its vector energy reserves, thereby extending its vector lifespan. This might be an explanation for the longer life span of *Borrelia*-infected *I. ricinus* and *I. persulcatus* ticks under laboratory conditions reported by Naumov [67]. Moreover, higher energy reserves in *Borrelia*-infected ticks allow more time for questing. Since *Borrelia-*infected ticks can move up and down the vegetation more often until their fat reserves are depleted, they survive longer and questing time is increased. In turn, longer questing time results in ticks harbouring *B. burgdorferi* spirochetes having more chances to find hosts, therefore making spirochete transmission more likely. In short, this situation illustrates that a pathogen may modify the phenotypic traits of its tick vector to enhance its transmission to another host: by increased tolerance to desiccation, increased tick survival under desiccating conditions, and higher energy reserves, eventually leading to increased opportunities for its vector to find a host.

## Conclusions

The idea that *B. burgdorferi* s.l. infection increases the opportunities that *I. ricinus* has to find a vertebrate host due to modified questing activity is particularly interesting. Faulde and Robbins [68] have reported that host-finding efficacy is increased in female *I. ricinus* ticks harbouring *B. burgdorferi* spirochetes. These authors have observed that *Borrelia* infection prevalence is higher in unfed ticks collected from clothing of volunteers than in unfed ticks collected directly from vegetation in the same forest in Germany. In the context of climate change, if weather conditions become warmer and drier (which is expected to favour *Borrelia*-infected *I. ricinus* ticks according to these findings), it can be surmised that the risk for human population to encounter ticks harbouring *B. burgdorferi* spirochetes, and therefore to be bitten by such individuals, might increase. As Lyme borreliosis is to date the most frequent tick-borne disease in the Northern hemisphere, accounting for approximately 85,500 new human cases annually, among which roughly 65,500 occur in Europe [69], we might expect this disease to become a greater public health concern if desiccating conditions become more pronounced and *Borrelia*-infected ticks are more likely to find hosts under such conditions. However, such suppositions might prove to be inaccurate in the future since Lyme borreliosis epidemiology does not depend solely on *I. ricinus* but rather on multiple parameters, notably *Borrelia* reservoir-host populations, which might be negatively influenced by climate change.
